# A Hierarchical Machine Learning–Based Framework for Clinical Decision Support in Foot Orthosis Prescription: Algorithm Development and Validation Study

**DOI:** 10.2196/92831

**Published:** 2026-09-22

**Authors:** Ji-Yong Jung, Wooyeol Yang, Jung-Ja Kim

**Affiliations:** 1 Division of Biomedical Engineering, College of Engineering, Jeonbuk National University, 567 Baekje-daero, Deokjin-gu, Jeonju, Jeonbuk-do, 54896, Republic of Korea, 82 63-270-4063, 82 63-270-2247; 2 Cyber Warfare Technology Research Center, Cyber Security Research Division, Electronics and Telecommunications Research Institute, Daejeon, Republic of Korea; 3 Research Center of Healthcare & Welfare Instrument for the Aged, Jeonbuk National University, Jeonju, Jeonbuk-do, Republic of Korea

**Keywords:** clinical decision support system, foot orthosis prescription, hierarchical machine learning, multilabel classification, gradient boosting, outpatient clinical data

## Abstract

**Background:**

Foot orthosis prescription is a complex clinical decision-making process that involves selecting and combining multiple structural, functional, and material components based on heterogeneous biomechanical information. In routine outpatient practice, detailed biomechanical assessments are often incomplete, creating substantial variability in prescription decisions, and limiting the applicability of conventional machine learning (ML) models that assume fixed feature availability.

**Objective:**

This study aimed to develop and evaluate a hierarchical ML-based clinical decision support framework for foot orthosis prescription that accommodates variable clinical information availability, supports multilabel prescription decisions, and incorporates safety-oriented recommendation strategies for routine outpatient practice.

**Methods:**

A retrospective observational study was conducted using 6462 visit-level clinical encounters collected from a single institution between 2015 and 2020. Orthotic prescription was formulated as a multilabel prediction task involving 15 prescription components. A hierarchical modeling framework was implemented, consisting of a basic decision level using routinely available demographic and alignment variables, and an advanced level incorporating subtalar joint inversion and eversion range of motion measurements when available. Tree-based gradient boosting models were evaluated using 5-fold stratified grouped cross-validation. Model performance was assessed using component-wise area under the receiver operating characteristic curve (AUROC), area under the precision-recall curve (AUPRC), positive predictive value (PPV), macro-averaged Hamming loss, Top-K hit, and coverage rates based on probabilities calibrated using internal grouped Platt scaling, and component-specific safety-oriented threshold calibration.

**Results:**

The hierarchical models demonstrated consistent predictive performance across both decision levels, although precision-recall–based analyses indicated greater variability for low-prevalence prescription components. The Level 4 and Level 8 models achieved mean component-wise AUROC values of 0.792 and 0.816, respectively, with macro-averaged Hamming loss of 0.113 and 0.111. Using component-specific Platt scaling within the 5-fold stratified grouped cross-validation framework, Top-K analysis demonstrated a Top-1 hit rate of 86.7% and a Top-3 hit rate of 97.4%. When all prescribed components were considered, Top-K coverage reached 71.7% at Top-3 and increased to 97.0% at Top-8. Safety-oriented threshold calibration was performed separately for each prescription component, selecting the threshold that achieved the highest sensitivity while maintaining a specificity of at least 95%. Under this component-specific calibration strategy, the aggregated operating profile achieved a microaveraged specificity of 96.00% with a microaveraged sensitivity of 28.71%. Age-stratified analyses revealed distinct feature-importance patterns between pediatric and adult populations.

**Conclusions:**

The proposed hierarchical clinical decision support framework supported multicomponent foot orthosis prescription under variable information availability commonly encountered in outpatient practice. By generating ranked component-wise recommendations and high-confidence threshold-calibrated outputs, the framework may support clinician decision-making in routine orthotic practice. Further prospective validation will be required to determine its clinical utility.

## Introduction

### Background and Clinical Motivation

Foot deformities such as pes planus, pes cavus, and in-toeing gait are prevalent across the lifespan and are frequently associated with pain, fatigue, and secondary musculoskeletal complications [1-4]. Customized foot orthoses are widely prescribed as a conservative intervention to support lower-limb alignment and modulate biomechanical loading during gait [5-8]. Through modulation of subtalar joint (STJ) mechanics and redistribution of ground reaction forces, orthoses can reduce excessive pronation, alleviate tendon strain, and mitigate the progression of conditions such as plantar fasciitis and knee osteoarthritis [5,7]. In younger populations, orthotic interventions are also commonly considered to support age-appropriate gait development and functional alignment during growth, further underscoring the need for prescription strategies that account for developmental stage and evolving biomechanical demands [9].

Despite their widespread clinical use, foot orthosis prescriptions remain highly individualized and are largely dependent on clinician experience and heterogeneous biomechanical assessments. Orthotic prescription rarely involves selecting a single standardized device; instead, clinicians typically prescribe combinations of structural modifications, functional components, and material-specific base designs tailored to a patient’s biomechanical profile, age, diagnosis, and functional demands [10-12]. This inherent combinatorial complexity makes orthotic prescription difficult to standardize and challenging to support using conventional rule-based or single-output decision frameworks.

In practice, the availability of detailed biomechanical measurements varies substantially in routine outpatient settings. Assessments such as joint range of motion (ROM) testing are often performed selectively based on clinical judgment, time constraints, and patient presentation. As a result, substantial interclinician variability exists in routine outpatient prescription practice, even among patients with similar clinical profiles. This complexity positions orthosis prescription as a multilabel clinical decision-making problem rather than a simple categorical classification task, highlighting the need for clinical decision support systems (CDSS) that can accommodate heterogeneous information availability and reflect routine clinical prescribing workflows [13].

### Computational Approaches and Informatics Challenges

Advances in machine learning (ML) have increasingly facilitated data-driven approaches to clinical decision support across diverse medical domains. In the field of foot biomechanics, early computational studies primarily relied on linear regression models and rule-based decision trees to examine associations between anatomical measurements and foot pathologies [14,15]. However, such approaches have limited capacity to capture nonlinear and high-dimensional interactions among biomechanical variables. More recent studies have demonstrated improved performance using ensemble methods and neural network-based classifiers for tasks such as foot type classification based on plantar pressure data, footprint images, or wearable sensor signals [16-18]. Nevertheless, most existing ML-based approaches have focused on automated diagnosis or classification tasks, rather than supporting therapeutic prescription decisions that require explicit selection of treatment components.

From an informatics perspective, orthosis prescription poses additional challenges that extend beyond conventional diagnostic modeling. Prescription decisions involve the concurrent selection of multiple interdependent design elements—including structural modifications, functional components, and material properties—making the problem inherently multilabel in nature. Moreover, orthotic decision-making must operate within the constraints of real-world clinical workflows, where the availability of biomechanical measurements varies substantially across patients and visits. In contrast, most prior ML-based models have been developed using curated datasets with complete and uniformly acquired measurements under standardized protocols [19,20]. These limitations restrict the translational applicability of existing models and underscore the need for decision support frameworks that can accommodate varying levels of clinical information while preserving clinically meaningful prescription patterns.

### Routine Outpatient Data Limitations and the Need for Hierarchical Decision Support

In routine outpatient clinical settings, comprehensive biomechanical assessments are often selectively performed or omitted due to time constraints, interrater variability, and institutional differences in outpatient workflows. As a result, electronic health records frequently contain selective measurement availability and partially observed biomechanical information, creating a substantial gap between idealized ML models and the conditions under which clinical decisions are actually made [20,21]. Consequently, a clinically implementable decision support system must not only achieve high predictive performance under controlled conditions but also demonstrate stable, interpretable behavior across varying levels of information availability encountered at the point of care.

To address these challenges, decision support architectures must explicitly account for the hierarchical nature of clinical data acquisition. Fundamental alignment measures, such as resting calcaneal stance position (RCSP), are routinely documented and serve as important considerations in orthotic design decisions [22]. In contrast, more detailed biomechanical assessments, including STJ inversion and eversion ROM, are typically obtained only in selected clinical contexts where additional evaluation is warranted [23,24]. Clinically, RCSP functions as a mandatory baseline assessment during initial evaluation, whereas STJ ROM represents higher-resolution biomechanical information acquired selectively to refine prescription decisions. This naturally creates a hierarchical structure of information availability in routine practice. Modeling strategies that assume uniform access to a fixed set of clinical variables are therefore poorly aligned with real-world workflows. Instead, approaches capable of adapting to multiple tiers of information availability are required to ensure the practical applicability and scalability of CDSS [25]. Collectively, these considerations motivated the development of the hierarchical modeling framework evaluated in the present study.

### Prior Work and Study Rationale

Existing studies have demonstrated the feasibility of computational approaches for supporting orthotic prescription decisions through component-level prediction models [26]. However, most of these studies were developed using relatively small and homogeneous datasets and focused on predicting a single prescription outcome. This simplified formulation does not adequately reflect real-world orthotic design, where multiple components are prescribed simultaneously to address complex biomechanical needs.

Large-scale retrospective analyses of custom foot orthoses have further shown that clinicians commonly prescribe diverse combinations of structural, functional, and material-specific components, with substantial variability associated with individual experience and institutional practice patterns [27]. Despite this complexity, few studies have explicitly formulated orthotic prescription as a multilabel prediction problem. Moreover, the performance of such models under the incomplete and heterogeneous data conditions encountered in routine outpatient practice remains largely unexplored. In particular, most existing models implicitly assume uniform availability of biomechanical measurements or rely on imputation strategies that do not reflect how data are selectively acquired in real-world clinical workflows. Consequently, their applicability is limited when advanced biomechanical assessments are unavailable, which is common in routine outpatient settings. These limitations highlight a critical gap between existing computational approaches and the realities of clinical orthosis prescription.

From a clinical safety perspective, omission of a necessary orthotic component may result in inadequate biomechanical correction and persistent or worsening symptoms [28,29]. Accordingly, decision support systems for orthosis prescription should emphasize high reliability and flexible recommendation strategies that assist rather than replace clinical judgment. Providing ranked recommendation shortlists is particularly important in multicomponent prescription tasks, where clinicians must evaluate multiple potentially relevant orthotic elements while balancing completeness and over-recommendation. In addition, decision support systems should communicate recommendations in a manner that supports clinician oversight and minimizes the risk of unnecessary component suggestions. These considerations highlight the need for clinically deployable decision support frameworks that can learn prescription patterns from routine outpatient data while supporting safe and reliable decision-making in everyday practice.

### Objective of This Study

In this study, we propose a hierarchical ML-based CDSS for foot orthosis prescription using a large-scale single-institution outpatient clinical dataset comprising 6462 visit-level clinical encounters collected during routine outpatient care. The system is explicitly designed to reflect variability in clinical information availability by adopting a hierarchical structure, ranging from a basic decision level based on routinely assessed alignment variables to an advanced level incorporating detailed STJ inversion and eversion range of motion measurements. A tree-based gradient boosting framework was employed to accommodate heterogeneous feature distributions and selective measurement availability commonly encountered in routine outpatient data.

The objectives of this study were threefold: (1) to evaluate the performance of hierarchical ML models in predicting 15 orthotic prescription components within a multilabel decision-making framework; (2) to investigate age-dependent differences in the relative importance of clinical variables guiding orthotic prescription; and (3) to evaluate the clinical utility of the proposed system under routine outpatient data conditions using calibrated Top-K analysis and safety-oriented threshold calibration.

## Methods

### Study Design, Setting, and Data Source

We conducted a retrospective observational study using routine outpatient clinical data collected during foot orthosis prescription at a single clinical institution. The dataset was derived from patients evaluated at a specialized podiatric clinic within the Department of Rehabilitation Medicine at Chungnam National University Hospital (Daejeon, Republic of Korea) between 2015 and 2020. All records originated from usual-care clinical documentation rather than from a controlled research protocol.

The final dataset comprised 6462 visit-level encounters from pediatric and adult patients presenting with foot deformities and lower-extremity alignment abnormalities such as pes planus, pes cavus, and in-toeing gait. Because examinations were performed as part of routine clinical workflows, the availability of detailed biomechanical measurements varied across visits. Demographic variables and core alignment measures were routinely documented, whereas STJ inversion and eversion ROM were obtained selectively based on clinical judgment, time constraints, and patient presentation. This workflow-driven variability in feature availability motivated the hierarchical design of the decision support framework. Each encounter was modeled as an independent decision point, reflecting routine outpatient orthosis prescription practice in which prescription decisions are based on the contemporaneous clinical presentation and available measurements during a single visit. Repeated encounters were identified using combined name, sex, and age information, and only 100 of 6355 patients (1.57%) contributed more than one encounter. Because some individuals contributed multiple encounters, patient-level grouped cross-validation was used during model development and evaluation to ensure that all encounters from the same patient were assigned exclusively to either the training or validation folds. All preprocessing, feature construction, hyperparameter optimization, model training, and probability calibration steps were confined within the training folds to minimize optimistic bias.

### Ethical Considerations

The study protocol was reviewed by the Institutional Review Board of Jeonbuk National University and deemed exempt because it involved secondary analysis of fully deidentified retrospective data (JBNU 2021-08-011). Informed consent was waived by the institutional review board, and all data were anonymized prior to analysis. No identifiable participant images were included in the manuscript or supplementary materials. The study was conducted in accordance with the Declaration of Helsinki and relevant ethical guidelines.

### Clinical Variables and Feature Sets

Predictor variables were defined to reflect biomechanical measures commonly used in routine orthosis prescription and were selected based on (1) clinical relevance to orthotic design decisions and (2) feasibility within standard outpatient workflows. Age was treated as a continuous variable, and sex was encoded as a binary categorical variable.

Primary diagnostic categories (eg, pes planus, pes cavus, and in-toeing gait) were not included as predictor variables because the modeling framework was designed to rely on objective biomechanical and alignment features that more directly reflect the functional characteristics relevant to orthotic prescription.

RCSP was selected as the core alignment variable because of its clinical prominence in orthotic decision-making. Bilateral RCSP values (left and right) were recorded in degrees during relaxed weight-bearing examinations and were treated as continuous predictors to preserve laterality. For advanced biomechanical characterization, bilateral STJ inversion and eversion range of motion, measured in degrees, were incorporated when available.

To support robust learning while preserving clinical interpretability, two derived RCSP features were computed within each encounter: RCSP sum (left+right), representing overall hindfoot alignment magnitude, and RCSP difference (absolute left–right difference), representing alignment asymmetry.

Two feature sets were defined a priori based on measurement availability. The Level 4 feature set included sex, age, bilateral RCSP measurements, RCSP sum, and RCSP difference. The Level 8 feature set comprised all Level 4 variables with the addition of bilateral STJ inversion and eversion ROM.

### Orthosis Prescription Labels and Multilabel Formulation

Orthosis prescriptions were represented as 15 binary component labels (P1-P15), reflecting routine clinical practice in which multiple orthotic elements are frequently prescribed in combination. Each label was encoded as 1 (prescribed) or 0 (not prescribed). Labels were additionally grouped into clinically meaningful categories (eg, structural modification, functional modification, base material, cushioning, and structural support) to facilitate interpretation, while preserving component-level granularity for modeling.

Because orthotic prescriptions frequently involve combinations of multiple components within a single encounter, prescription was formulated as a multilabel prediction problem. For model development and evaluation, each prescription component was modeled as an independent binary classification task. Overall multilabel performance and clinical utility were assessed by aggregating component-level predictions using appropriate multilabel metrics. The 15 prescription components and their associated clinical categories are summarized in Table 1.

**Table 1.** Orthotic prescription components and their clinical categories used as multilabel outcomes.

Label	Orthotic component name	Clinical category
P1	Forefoot extension	Structural modification
P2	Poron base material	Base material
P3	Multidensity foam	Base material
P4	Cork-based foundation	Base material
P5	Polyethylene base material	Base material
P6	Metatarsal dome	Pressure redistribution
P7	Heel Poron cushion	Pressure redistribution
P8	Medial inverted balance posting	Structural support
P9	Medial forefoot flange	Structural modification
P10	Lateral flange	Structural modification
P11	Gait plate	Functional modification
P12	Reverse gait plate	Functional modification
P13	UCBL^a^	Structural support
P14	Navicular support	Structural support
P15	Kinetic wedge	Functional modification

a UCBL: University of California Biomechanics Laboratory orthosis.

### Missing Data Handling and Hierarchical Evaluation Strategy

**Figure 1.**
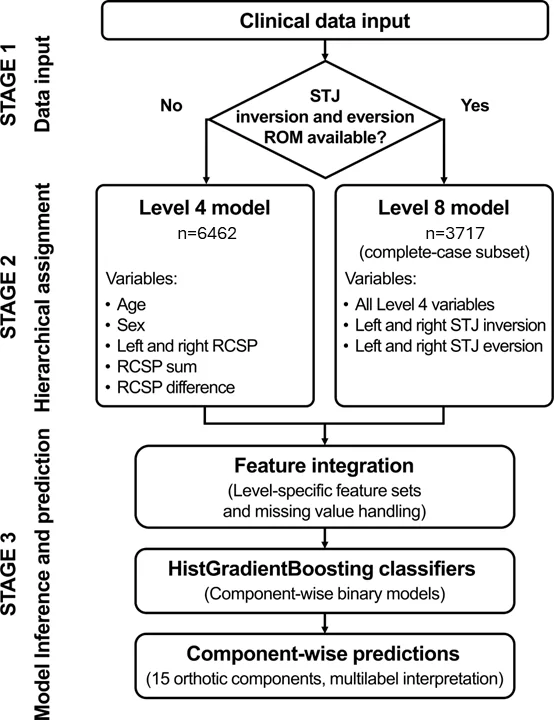
Overview of the proposed hierarchical clinical decision support framework for foot orthosis prescription. RCSP: resting calcaneal stance position; ROM: range of motion; STJ: subtalar joint.

Missingness in biomechanical variables reflected selective measurement practices in routine clinical care rather than random acquisition failures. In particular, STJ inversion and eversion ROM were not uniformly recorded across encounters because these assessments were performed selectively according to clinical judgment. An overview of the hierarchical clinical decision support framework is illustrated in Figure 1.

A hierarchical evaluation strategy was adopted based on measurement availability. Encounters with available STJ inversion and eversion ROM were evaluated using the Level 8 model, whereas encounters without these measurements were evaluated using the Level 4 model. As a result, Level 8 performance reflects a complete-case subset analysis restricted to encounters with available STJ ROM measurements. For direct comparison between the two hierarchical levels, both Level 4 and Level 8 were additionally evaluated using the same complete-case subset.

Within each predefined feature set, remaining missing values were retained as not a number (NaN) and handled using the native missing-value processing capability of the tree-based algorithms used in this study. This approach avoids ambiguity between missing observations and valid biomechanical measurements.

### Model Development and Implementation

To benchmark model performance, several tree-based and ensemble learning algorithms were comparatively evaluated, including HistGradientBoosting (HGB), Extreme Gradient Boosting (XGBoost), Light Gradient Boosting Machine (LightGBM), and a soft-voting ensemble combining the three models. All comparator models were trained using the same feature sets, cross-validation framework, and component-wise binary classification strategy to ensure fair comparison across hierarchical decision levels. Comparative performance was assessed using mean component-wise area under the receiver operating characteristic curve (AUROC) and macro-averaged Hamming loss. HGB was selected as the final architecture because it consistently achieved strong performance across hierarchical levels while naturally accommodating the heterogeneous and partially observed characteristics of the clinical dataset.

For each prescription component (P1—P15), a separate binary classifier was trained within each hierarchical level using the corresponding feature set. Sex was encoded as a binary categorical variable, and continuous predictors were retained in their original scales without normalization, consistent with the properties of tree-based learning methods. No explicit class weighting was applied during model training.

Hyperparameters for each prescription component and hierarchical level were optimized using Optuna with 5-fold stratified grouped cross-validation. Candidate parameter combinations were evaluated according to the mean out-of-fold AUROC obtained from the grouped cross-validation folds, and the configuration achieving the highest mean AUROC was selected as the final component-specific parameter set. Optimization was conducted over predefined search spaces for max_iter, max_depth, learning_rate, l2_regularization, max_bins, and min_samples_leaf, with parameter-specific sampling distributions (Table S3 in Multimedia Appendix 1). The resulting optimized parameter settings are reported in Table S4 in Multimedia Appendix 1. Patient-level grouping was maintained throughout hyperparameter optimization, and a fixed random seed (42) was applied to ensure reproducibility.

### Evaluation Metrics and Cross-Validation

Model discrimination was evaluated using AUROC, computed separately for each prescription component. Performance across components was summarized using the mean component-wise AUROC. In addition, the area under the precision-recall curve (AUPRC) and positive predictive value (PPV) were calculated for each prescription component. PPV was calculated using binary predictions generated with the default probability threshold of 0.5 and was included to provide a prevalence-sensitive measure of positive prediction performance.

Evaluation was conducted using 5-fold stratified grouped cross-validation. Patient identifiers were used as grouping variables to ensure that all encounters from the same patient were assigned exclusively to either the training or validation folds, with class distributions preserved within each fold. All preprocessing, feature construction, hyperparameter application, model training, and probability calibration were performed exclusively within the training folds and subsequently applied to the corresponding validation folds to prevent information leakage.

To quantify overall multilabel misclassification, component-wise Hamming loss was calculated using binary predictions generated with the default probability threshold of 0.5 and then macro-averaged across the 15 prescription components. In addition, clinical utility was evaluated using Top-K analysis and safety-oriented threshold calibration.

For safety-oriented threshold calibration, candidate probability thresholds from 0.01 to 0.99 were evaluated separately for each prescription component using out-of-fold predictions. For each classifier, the operating threshold was selected as the value achieving the highest sensitivity while maintaining a specificity of at least 95%.

For Top-K analysis, calibrated out-of-fold probabilities were generated within an outer 5-fold stratified grouped cross-validation framework. Within each outer training fold, each component-specific HGB classifier was calibrated using Platt scaling with an additional internal stratified grouped cross-validation procedure. Patient-level grouping was maintained in both the outer and internal cross-validation procedures to ensure that encounters from the same patient were not shared across model-training, calibration, and outer validation subsets. The calibrated model was then applied to the corresponding outer validation fold, and the resulting probabilities were aggregated across folds to obtain complete out-of-fold predictions for all encounters. For each encounter, the calibrated predicted probabilities of the 15 prescription components were ranked in descending order to generate component-level recommendations. Top-K hit rate was defined as the proportion of encounters in which at least one clinician-prescribed component was included among the Top-K recommendations. Top-K coverage was defined as the mean proportion of clinician-prescribed components captured within the Top-K recommendations for each encounter, accounting for encounters with multiple prescribed components. 95% CIs for Top-K metrics were estimated using 1000 bootstrap resamples.

To characterize potential selection bias, baseline characteristics of the full cohort and the Level 8 complete-case subset were compared using independent *t* tests for continuous variables and chi-square tests for categorical variables. A 2-sided *P*<.05 was considered statistically significant.

### Feature Importance Analysis

Feature importance was assessed using permutation importance analysis. For each predictor, values were randomly permuted and the resulting change in model performance was calculated relative to the unpermuted model. Permutation was repeated 5 times, and mean importance values were used for visualization. Positive values indicate that permutation reduced model performance, whereas negative values indicate negligible or unstable predictive contribution and should not be interpreted as evidence of inverse association. Age-stratified feature importance analysis was performed within the Level 8 complete-case subset (n=3717).

## Results

### Overall Performance of Hierarchical Models (AUROC and Hamming Loss)

**Figure 2.**
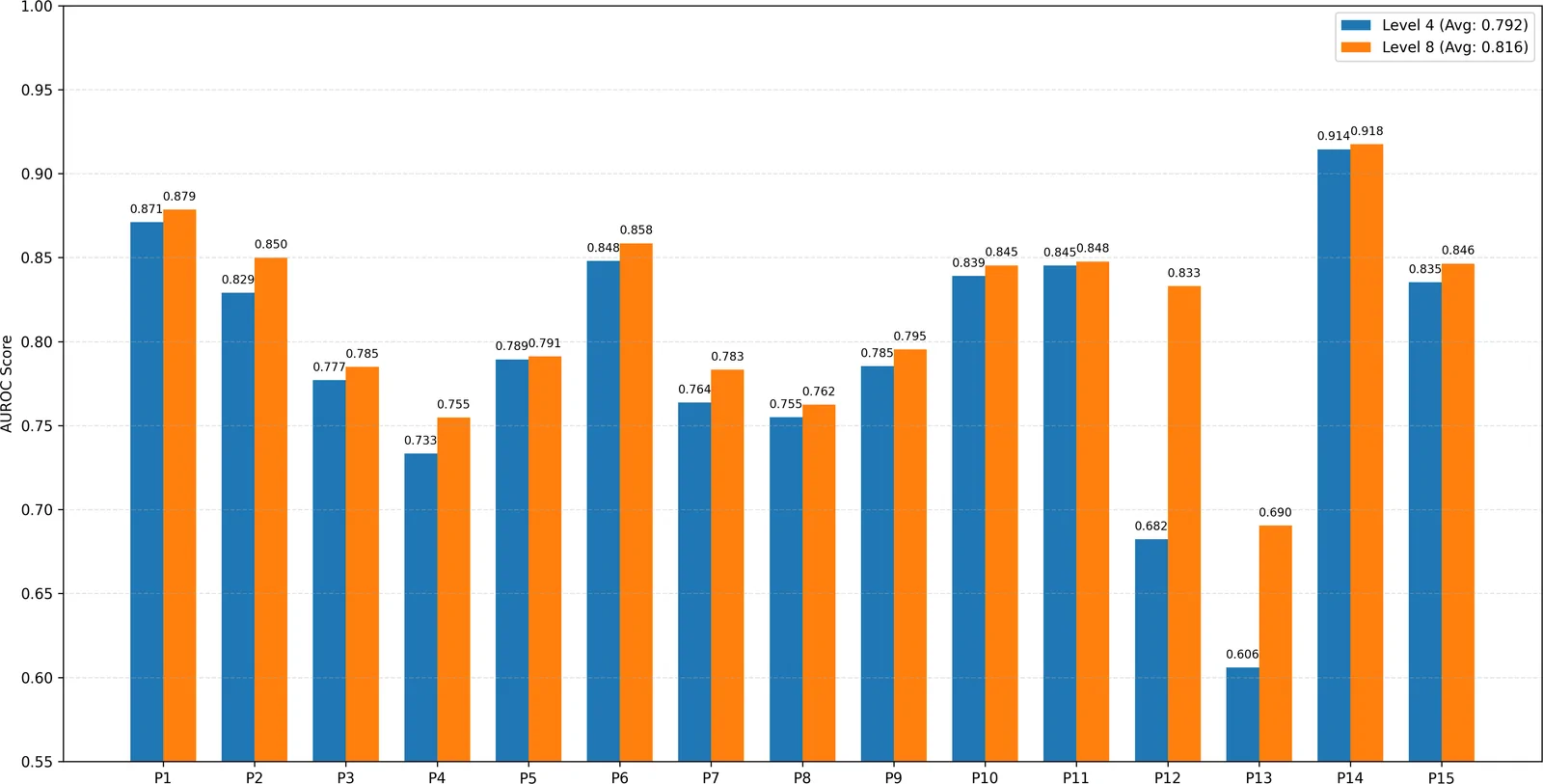
Component-wise AUROC (area under the receiver operating characteristic curve) comparison between the Level 4 and Level 8 hierarchical gradient boosting–based models evaluated on the same complete-case subsets (n=3717).

Based on model comparison, the Optuna-tuned HGB model was selected for subsequent analyses. To enable a direct comparison between hierarchical levels, both models were evaluated using the same complete-case subset (n=3717). As illustrated in Figure 2, the mean component-wise AUROC increased from 0.792 to 0.816, while the macro-averaged Hamming loss decreased from 0.113 to 0.111. These results indicate that the improved performance of the Level 8 model was associated with the additional STJ inversion and eversion ROM measurements rather than differences in cohort composition.

Baseline characteristics of the Level 8 complete-case subset compared with the full cohort are provided in Table S2 in Multimedia Appendix 1. The Level 8 subset differed from the full cohort in several demographic, alignment, and prescription characteristics, suggesting that advanced biomechanical measurements were obtained in a clinically selective subgroup.

As summarized in Table 2, component-wise analysis revealed heterogeneous predictive performance across the 15 prescription labels. Structural elements, such as navicular support (P14; AUROC=0.918) and forefoot extension (P1; AUROC=0.879), demonstrated robust discriminative ability in the Level 8 model. Performance improvements were observed for most prescription components following the inclusion of STJ inversion and eversion ROM measurements, whereas gains were limited or absent for a small number of components, particularly those with very low prevalence.

**Table 2.** Baseline prevalence and component-wise performance (AUROC^a^, AUPRC^b^, and PPV^c^) of the Level 4 and Level 8 hierarchical models evaluated using the same complete-case subset (n=3717), together with macro-averaged Hamming loss^d^.

Label	Component	N (%)	Level 4, mean (SD)			Level 8, mean (SD)		
			AUROC	AUPRC	PPV	AUROC	AUPRC	PPV
P1	Forefoot extension	3248 (87.4)	0.871 (0.023)	0.972 (0.009)	0.899 (0.015)	0.879 (0.021)	0.974 (0.010)	0.903 (0.014)
P2	Poron base material	537 (14.4)	0.829 (0.010)	0.497 (0.020)	0.643 (0.054)	0.850 (0.017)	0.503 (0.022)	0.610 (0.053)
P3	Multidensity foam	2226 (59.9)	0.777 (0.024)	0.808 (0.029)	0.721 (0.027)	0.785 (0.021)	0.815 (0.026)	0.733 (0.029)
P4	Cork-based foundation	878 (23.6)	0.733 (0.017)	0.446 (0.012)	0.617 (0.033)	0.755 (0.026)	0.458 (0.022)	0.527 (0.050)
P5	Polyethylene base material	454 (12.2)	0.790 (0.021)	0.390 (0.025)	0.553 (0.138)	0.791 (0.025)	0.382 (0.041)	0.555 (0.161)
P6	Metatarsal dome	392 (10.5)	0.848 (0.019)	0.346 (0.047)	0.000 (0.000)	0.858 (0.021)	0.356 (0.048)	0.380 (0.232)
P7	Heel poron cushion	2406 (64.7)	0.763 (0.015)	0.827 (0.009)	0.753 (0.019)	0.783 (0.012)	0.848 (0.010)	0.767 (0.025)
P8	Medial inverted balance posting	629 (16.9)	0.755 (0.025)	0.363 (0.034)	0.486 (0.060)	0.762 (0.021)	0.390 (0.031)	0.571 (0.063)
P9	Medial forefoot flange	152 (4.1)	0.785 (0.048)	0.117 (0.021)	0.000 (0.000)	0.795 (0.036)	0.124 (0.021)	0.000 (0.000)
P10	Lateral flange	100 (2.7)	0.839 (0.045)	0.275 (0.117)	0.750 (0.440)	0.845 (0.049)	0.308 (0.105)	1.000 (0.548)
P11	Gait plate	450 (12.1)	0.845 (0.028)	0.421 (0.117)	0.559 (0.205)	0.848 (0.023)	0.427 (0.108)	0.587 (0.218)
P12	Reverse gait plate	12 (0.3)	0.682 (0.074)	0.005 (0.005)	0.000 (0.000)	0.833 (0.127)	0.021 (0.046)	0.000 (0.000)
P13	UCBL^e^	26 (0.7)	0.606 (0.169)	0.016 (0.018)	0.000 (0.000)	0.690 (0.152)	0.015 (0.012)	0.000 (0.000)
P14	Navicular support	419 (11.3)	0.914 (0.014)	0.644 (0.054)	0.701 (0.088)	0.918 (0.012)	0.655 (0.042)	0.718 (0.065)
P15	Kinetic wedge	72 (1.9)	0.835 (0.060)	0.084 (0.040)	0.000 (0.000)	0.846 (0.062)	0.080 (0.029)	0.000 (0.000)
Mean	Mean component-wise AUROC	—^f^	0.792 (0.078)	0.414 (0.299)	0.445 (0.340)	0.816 (0.057)	0.424 (0.299)	0.490 (0.341)
Macro-averaged	Hamming loss	—	0.113	—	—	0.111	—	—

a AUROC: area under the receiver operating characteristic curve.

b AUPRC: area under the precision-recall curve.

c PPV: positive predictive value.

d PPV was calculated using binary predictions generated with the default probability threshold of 0.5. Values are presented as mean (SD), where SD represents variability across the 5 grouped cross-validation folds. For low-prevalence components, SD values may be large relative to the mean, and point estimates should be interpreted with caution.

e UCBL: University of California Biomechanics Laboratory orthosis.

f Not applicable.

Within the matched complete-case subset (n=3717), prescription prevalence varied substantially across components, ranging from 12/3717 (0.3%) for P12 to 3248/3717 (87.4%) for P1. To further evaluate performance under class imbalance, AUPRC and PPV were additionally assessed. Mean component-wise AUPRC increased slightly from 0.414 for the Level 4 model to 0.424 for the Level 8 model, while mean PPV increased from 0.445 to 0.490. Despite acceptable AUROC values, several low-prevalence prescription components exhibited low AUPRC and PPV. For example, P12 achieved an AUROC of 0.833 in the Level 8 model but an AUPRC of only 0.021 and a PPV of 0.000. These findings indicate that discrimination performance should be interpreted alongside prevalence-sensitive metrics, particularly for rare prescription components. Fold-to-fold variability was also greater for several low-prevalence components, especially for PPV estimates.

To quantify overall multilabel misclassification, macro-averaged Hamming loss was calculated across the 15 prescription components. The Level 4 model yielded a macro-averaged Hamming loss of 0.113, which decreased slightly to 0.111 in the Level 8 model, indicating improved overall multilabel prediction performance following the inclusion of STJ measurements.

### Model Comparison and Selection

To determine the optimal learning algorithm under a fair matched-cohort comparison, several tree-based ensemble methods were evaluated using the same complete-case subset for both hierarchical levels. Model performance was evaluated using the mean component-wise AUROC across the 15 prescription components for both hierarchical levels within the matched complete-case subset.

As summarized in Table 3, the Optuna-tuned HGB model achieved the highest mean component-wise AUROC among all evaluated algorithms (Level 4: 0.792; SD 0.078; Level 8: 0.816; SD 0.057). Compared with the default HGB model, Optuna-based hyperparameter optimization resulted in modest improvements, particularly for the Level 8 model. Consequently, the Optuna-tuned HGB model was selected for all subsequent analyses.

**Table 3.** Comparison of mean component-wise AUROC^a^ across learning algorithms for the Level 4 and Level 8 hierarchical models evaluated using the same complete-case subset (n=3717)^b^.

Model	Level 4	Level 8
XGBoost^c^	0.790 (0.083)	0.811 (0.064)
LightGBM^d^	0.789 (0.085)	0.808 (0.064)
HGB^e^	0.790 (0.079)	0.809 (0.058)
Soft-voting ensemble (XGBoost + LightGBM + HGB)	0.786 (0.083)	0.806 (0.065)
Optuna-tuned HGB	0.792 (0.078)	0.816 (0.057)

a AUROC: area under the receiver operating characteristic curve.

b Values are presented as mean (SD), where SD represents variability across the 15 prescription components.

c XGBoost: Extreme Gradient Boosting.

d LightGBM: Light Gradient Boosting Machine.

e HGB: HistGradientBoosting.

### Age-Dependent Predictive Patterns in Orthotic Prescription

**Figure 3.**
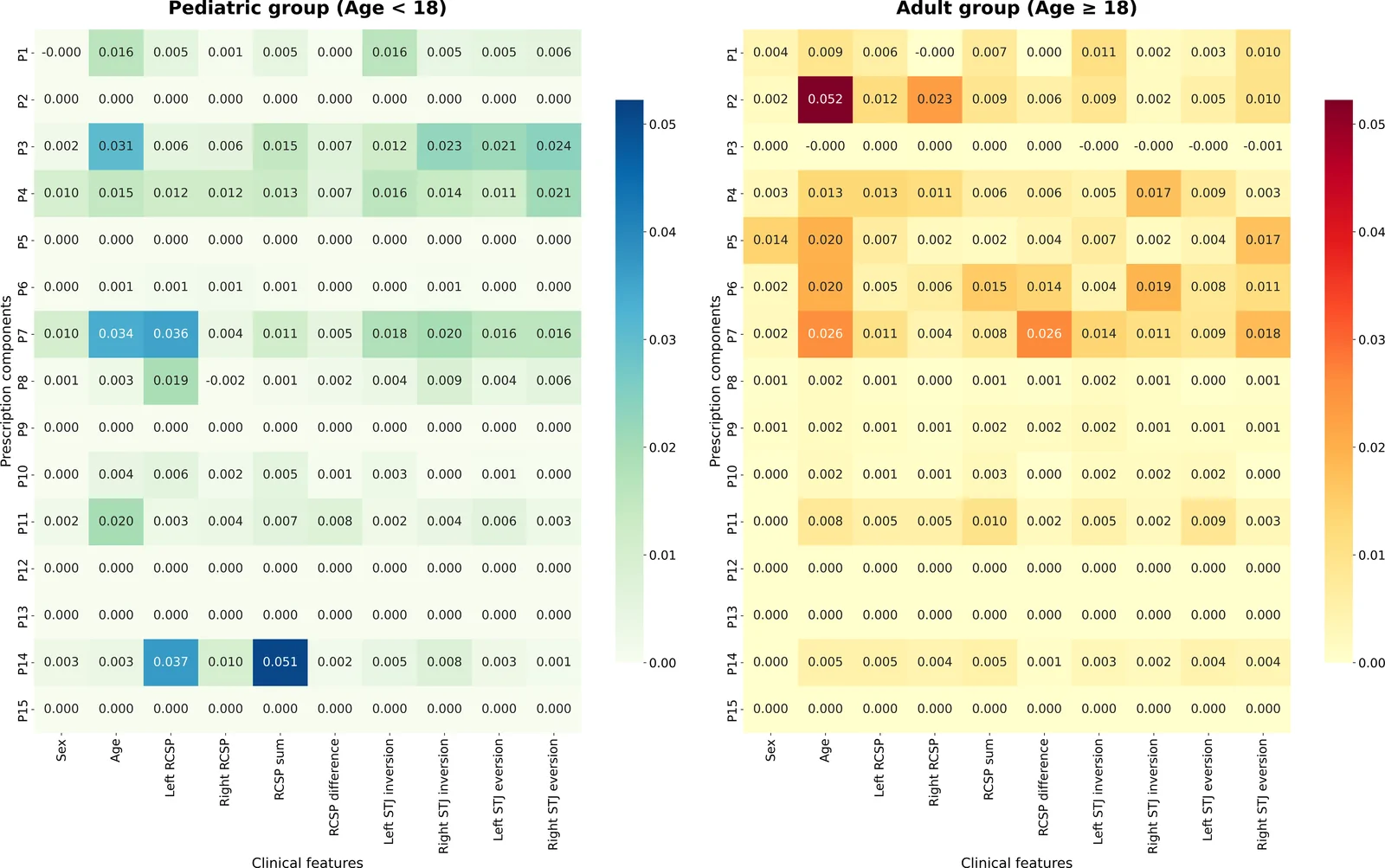
Age-stratified variable importance for orthotic prescription components. RCSP: resting calcaneal stance position; STJ: subtalar joint.

To investigate developmental differences in predictive feature importance patterns, the Level 8 complete-case subset was stratified into pediatric (age <18) and adult (age ≥18) groups. While overall predictive performance remained stable, the relative importance of clinical variables exhibited distinct, age-dependent profiles, as illustrated in Figure 3.

In the pediatric subgroup, prediction patterns were primarily associated with alignment-related factors, reflecting the importance of hindfoot correction during developmental stages. Age contributed meaningfully to several components (P1, P3, P7, and P11) but did not uniformly dominate decision-making, with the strongest contributions observed for P7 and P3. Hindfoot alignment measures, particularly RCSP-derived features, showed pronounced importance for alignment-related structural support components, most notably navicular support (P14), where RCSP sum exhibited the highest importance. In contrast, STJ inversion and eversion ROM exhibited generally modest importance across most pediatric components, with measurable contributions limited to select prescriptions such as forefoot extension (P1) and cushioning-related components (P4 and P7). Several low-prevalence components (P2, P5, P6, P12, P13, and P15) showed near-zero importance values across variables, suggesting limited learnable signal within the pediatric subset. Overall, alignment-related variables showed greater predictive importance than STJ mobility measures in the pediatric subgroup.

Adult orthotic prescriptions exhibited a distinct predictive pattern. Age demonstrated a dominant contribution for several prescriptions, most prominently Poron base material (P2), and also contributed meaningfully to selected cushioning and pressure-redistribution components, including the metatarsal dome (P6) and heel Poron cushioning (P7), while alignment- and mobility-related variables provided complementary predictive information across multiple prescription labels. Alignment-related measures (bilateral RCSP and RCSP-derived features) remained important across multiple prescription components. For some labels, their contributions were comparable to those of age (eg, RCSP sum for P6). STJ inversion and eversion ROM demonstrated modest, component-specific contributions rather than broad dominance, with the most visible signals appearing in selected cushioning- and foundation-related prescriptions (P4 and P7). Notably, for heel cushioning (P7), RCSP difference showed a relatively large contribution to model prediction compared with several other variables. Overall, adult prescription patterns were associated with a broader combination of age, alignment, and mobility-related variables.

### Clinical Utility of Top-K Analysis and Safety-Oriented Calibration

**Figure 4.**
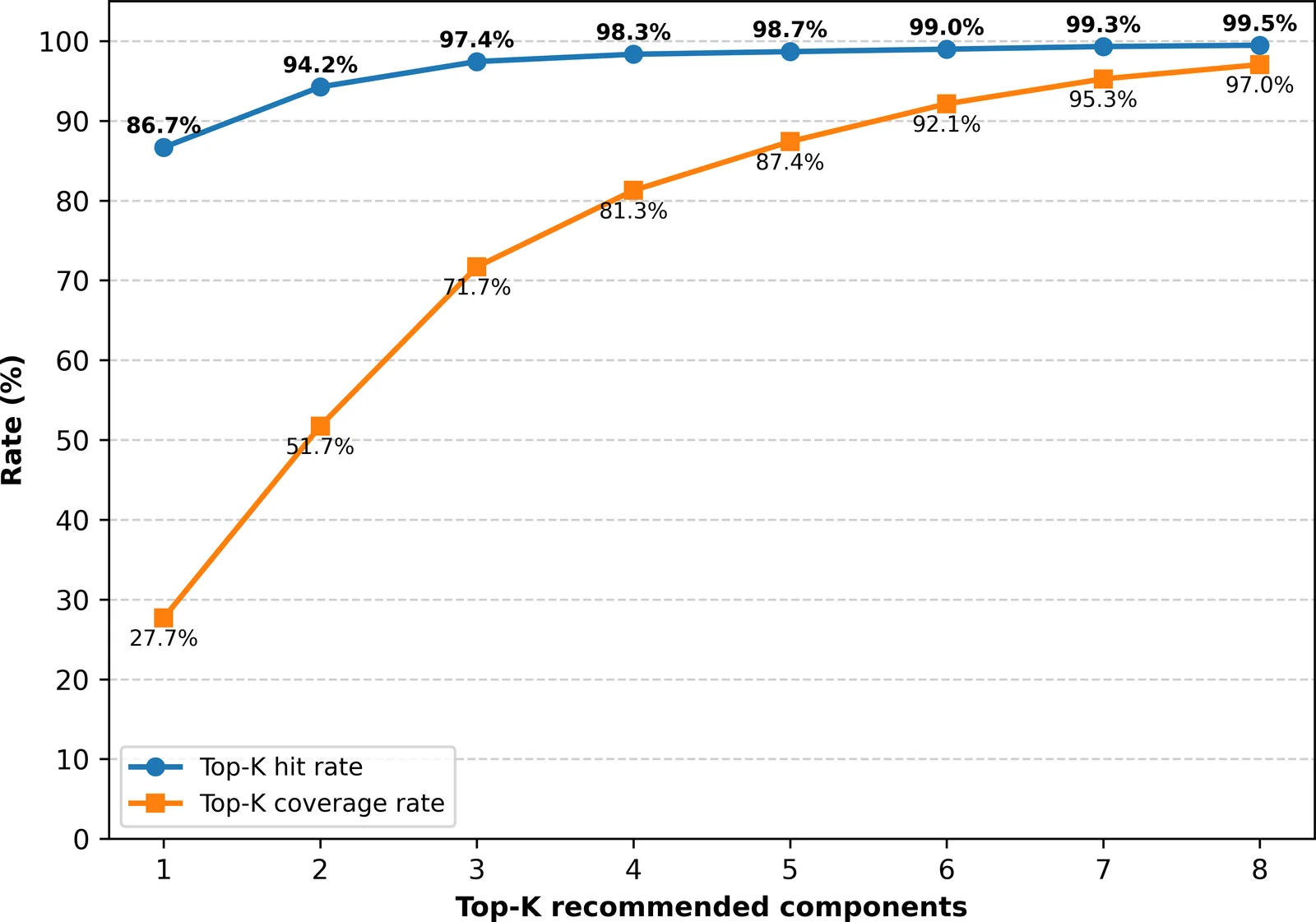
Top-K hit rate and Top-K coverage rate for clinician-prescribed orthotic components among model-ranked recommendations.

Clinical utility was evaluated using Top-K analysis and safety-oriented threshold calibration (Figure 4 and Table 4). Unlike traditional categorical accuracy, Top-K analysis evaluates whether clinician-prescribed components are included among the model’s highest-ranked recommendations and the extent to which these components are captured within a given recommendation set, reflecting a clinician-in-the-loop workflow.

**Table 4.** Aggregated confusion matrix obtained using component-specific safety-oriented threshold calibration across the 15 orthotic prescription components^a^.

Actual prescription	Predicted no	Predicted yes
No	96.00%	4.00 %
Yes	71.29 %	28.71 %

a Values are row-normalized percentages. For each prescription component, the operating threshold was selected to maximize sensitivity while maintaining a specificity of at least 95%. The resulting microaveraged specificity and sensitivity were 96.00% and 28.71%, respectively.

As illustrated in Figure 4, the Top-1 hit rate was 86.7% (95% CI 85.6‐87.8) and increased to 97.4% (95% CI 96.9‐97.9) at Top-3, indicating that a compact recommendation set frequently included at least one clinician-prescribed component. The hit rate continued to increase gradually, reaching 99.5% (95% CI 99.2‐99.7) at Top-8. Top-K coverage increased more gradually, reaching 71.7% (95% CI 70.9‐72.4) at Top-3 and 97.0% (95% CI 96.7‐97.4) at Top-8 when all prescribed components were considered. Top-K rankings were generated using probability-calibrated out-of-fold predictions obtained through nested grouped cross-validation, improving comparability across prescription components while avoiding probability calibration on the same data used to train the corresponding base classifiers.

To assess clinical safety, safety-oriented threshold calibration was performed separately for each prescription component. As shown in Table 4, the aggregated confusion matrix demonstrated a microaveraged specificity of 96.00%, corresponding to a false-positive rate of 4.00%. The resulting microaveraged sensitivity of 28.71% reflects the conservative operating profile adopted for safety-oriented decision support. Component-specific thresholds, sensitivities, and specificities for all 15 prescription labels are provided in Multimedia Appendix 1 Table S1. Although several low-prevalence prescription components maintained high specificity, their sensitivities remained relatively low, indicating reduced detection of rare prescription labels under the conservative calibration strategy.

Table 5 summarizes the age-stratified performance of the component-specific safety-oriented threshold calibration strategy in the Level 8 complete-case subset (n=3717). Using the same component-specific thresholds, the pediatric subgroup (Age <18, n=2570) achieved a microaveraged sensitivity of 0.243 and a specificity of 0.967, whereas the adult subgroup (Age ≥18, n=1147) achieved a microaveraged sensitivity of 0.368 and a specificity of 0.943. Although the adult subgroup demonstrated higher sensitivity, both subgroups maintained specificity above 94%, suggesting that the safety-oriented calibration objective was preserved across age groups.

**Table 5.** Age-stratified safety-oriented threshold calibration results for the Level 8 complete-case subset (n=3717).

Age group	Sensitivity	Specificity
Pediatric (Age <18, n=2570)	0.243	0.967
Adult (Age ≥18, n=1147)	0.368	0.943

## Discussion

### Principal Findings

The proposed hierarchical CDSS demonstrated stable overall performance across both decision levels, though precision-recall–based analyses revealed substantial performance heterogeneity, particularly among low-prevalence prescription components. These results highlight the importance of interpreting AUROC together with prevalence-sensitive metrics when evaluating multilabel clinical prediction models under class imbalance. Overall, the framework appeared more reliable for moderate- and high-prevalence prescription components, whereas predictive performance for rare prescription components remains limited despite acceptable AUROC values in some cases.

Notably, the model achieved a Top-1 hit rate of 86.7% and a Top-3 hit rate of 97.4%, indicating that a compact recommendation shortlist frequently included at least one clinician-prescribed component. Top-K coverage increased more gradually, reaching 71.7% at Top-3 and 97.0% at Top-8 when all prescribed components were considered. This divergence between hit rate and coverage indicates that a concise shortlist may provide efficient initial decision support, whereas capturing the full set of prescribed orthotic components in multicomponent prescriptions may require broader recommendation sets.

The proposed framework was designed to prioritize clinical safety through a conservative operating profile. Component-specific thresholds were selected to maximize sensitivity while maintaining a specificity of at least 95% for each prescription component, thereby reducing the likelihood of unnecessary component recommendations [30,31]. The resulting aggregated operating profile achieved a microaveraged specificity of 96.00%. Although this strategy resulted in a lower microaveraged sensitivity of 28.71%, this trade-off reflects the role of the CDSS as a clinician-supervised decision support tool rather than an autonomous prescription system.

The hierarchical architecture remained robust despite the variability of routine outpatient data. Although advanced biomechanical measurements such as STJ ROM were not consistently available, the framework maintained stable predictive performance by adapting to the information available at the time of prescription. Using the same complete-case subset (n=3717), the mean component-wise AUROC increased from 0.792 in the Level 4 model to 0.816 in the Level 8 model, indicating incremental predictive value from STJ inversion and eversion measurements. This incremental pattern remained consistent under patient-level grouped cross-validation, suggesting that the observed difference was not attributable to repeated patient encounters. Although the magnitude of improvement varied across prescription components, most components showed stable or improved discrimination following the inclusion of STJ measurements. Despite this component-specific variability, the overall findings suggest that the framework may provide clinically useful decision support even when comprehensive biomechanical assessments are not always feasible [32].

Age-stratified analyses identified distinct age-related differences in feature importance patterns. Variable importance profiles differed between pediatric and adult groups, with pediatric prescription patterns showing stronger associations with alignment-related measures, whereas adult prescription patterns demonstrated relatively greater contributions from age for selected prescription components. These patterns should be interpreted as model-derived predictive associations rather than evidence of causal relationships in prescription decision-making and are broadly consistent with clinical expectations [33].

### Comparison With Previous Work

Previous studies in foot orthotics and rehabilitation have demonstrated the feasibility of applying ML techniques to biomechanical assessment and gait analysis. However, most prior work has focused on tasks adjacent to prescription, such as diagnostic classification or outcome prediction, rather than directly supporting the complex, multicomponent decision-making process involved in orthotic prescription.

Several studies have explored ML-based insole or footwear recommendation by formulating the task as a single-output classification problem in which each patient is assigned to one predefined insole type or footwear category. For example, Kim et al [34] proposed a deep learning-based framework for prescribing customized insoles for patients with foot pain, demonstrating that convolutional neural network models could map plantar pressure patterns and clinical features to predefined insole categories with promising predictive performance. Similarly, Rajagopal et al [35] developed a ML pipeline that combined automated foot condition detection and classification with subsequent footwear recommendation. Although these studies demonstrated the clinical potential of data-driven insole and footwear recommendation, the prescription task was ultimately reduced to selecting a single mutually exclusive category. This formulation does not reflect routine clinical orthotic practice, where clinicians assemble combinations of multiple structural and material components tailored to individual patient needs [36]. To better reflect routine orthotic practice, the present study formulated prescription as a multilabel decision-making problem involving 15 orthotic components.

Another important body of research has focused on automating biomechanical measurement using ML. For example, Mun and Choi [37], and Liao et al [38] proposed deep learning–based approaches to estimate plantar pressure distributions and derive clinically relevant indices from sensor data, while Dindorf et al [39] developed scalable frameworks for automated plantar pressure analysis across multicenter datasets. Although these studies represent important methodological advances in biomechanical assessment, their primary contribution lies in measurement automation rather than in translating biomechanical information into actionable prescription decisions. Related investigations have emphasized outcome prediction following orthotic intervention, such as predicting gait speed or modeling postintervention pressure changes [40,41]. While clinically meaningful, these approaches focus on downstream effects of intervention rather than the upstream decision of how orthotic components should be selected and combined at the time of prescription [42].

A separate line of research has framed foot-related ML tasks as diagnostic or classification problems. Studies by Agrawal et al [43], Xu et al [44], and Jun et al [45] demonstrated the utility of plantar pressure, inertial, and multimodal data for classifying foot pathology or abnormal gait patterns. However, these models do not address the combinatorial nature of orthotic design, in which multiple interacting components are prescribed simultaneously [46].

In the rehabilitation domain, ML-based studies have also examined ankle-foot orthosis (AFO)–related decision-making. Prior work has focused either on predicting the need for an AFO or on optimizing control strategies for powered AFO systems [47,48]. Although clinically relevant, these approaches primarily address high-level indication or intervention execution rather than supporting detailed configuration of orthotic design elements at the prescription stage.

A notable distinction between the present work and many previous ML studies is the explicit framing of the system as a clinical decision support tool [49,50]. While predictive performance is often emphasized, comparatively less attention has been given to workflow integration, uncertainty communication, and clinician interaction. The Developmental and Exploratory Clinical Investigations of Decision support systems driven by AI (DECIDE-AI) reporting guideline highlights the importance of evaluating AI-enabled decision support not only on discrimination metrics but also on intended use, workflow fit, and early-phase clinical evaluation design [51]. Consistent with this perspective, previous studies have shown that clinician responses to AI recommendations depend strongly on presentation format and explanation style [52-54].

In contrast to these prior approaches, the present study was intentionally designed as a CDSS rather than an automated prescription tool. Orthotic prescription was formulated as a multilabel, component-based decision-making problem because clinical prescription involves combining multiple orthotic elements rather than selecting a single device category. However, each prescription component was modeled as an independent binary classification task, and therefore statistical dependencies among prescription labels were not explicitly modeled. To improve cross-label comparability, probability calibration was applied before generating ranked recommendations. By generating probabilistic, component-wise recommendations, the proposed framework narrows a complex decision space while preserving clinician oversight in the final prescription process.

Another distinguishing feature of this study is its hierarchical architecture, which was designed to reflect routine clinical workflows in which advanced biomechanical measurements are not always available. Rather than requiring a fixed set of inputs, the framework adapts to available clinical information by relying on alignment-based variables when advanced measurements are unavailable and incorporating mobility-related variables when such measurements are present. This flexible structure was intended to accommodate heterogeneous data conditions commonly encountered in outpatient orthotic practice and represents a workflow-oriented alternative to previous biomechanical, diagnostic, and outcome-focused ML approaches.

### Clinical Implications

In clinical practice, the proposed CDSS can function as a safety-oriented and workflow-compatible support tool that augments clinician decision-making. By presenting a concise shortlist of high-probability components, the system may reduce cognitive burden and support more consistent prescription practices among clinicians with varying levels of experience. It may also serve as a bridge between junior and experienced clinicians, potentially contributing to more standardized orthotic prescription practices [46,55]. For less experienced clinicians, Top-K recommendations provide structured guidance grounded in historical prescription patterns. For experienced clinicians, the system functions as a safety checklist that helps reduce inadvertent omissions in time-constrained settings while preserving professional autonomy.

A safety-oriented operating profile was adopted because unnecessary orthotic components may impose additional cost, complexity, or burden without clear clinical benefit. Component-specific threshold calibration was therefore applied to minimize the risk of false-positive recommendations. Although this strategy resulted in lower overall sensitivity, the resulting trade-off is consistent with the intended role of the CDSS as a clinician-supervised decision support tool designed to provide high-confidence recommendations rather than autonomous prescription decisions. Threshold-calibrated recommendations and Top-K recommendations serve complementary purposes. Whereas threshold-calibrated outputs emphasize high-confidence recommendations with minimal false positives, the Top-K framework provides a broader ranked shortlist that supports clinician review and decision-making during the prescription process.

Beyond clinician-facing decision support, the proposed CDSS may also function as a communication aid during patient consultations [56]. By providing probabilistic outputs linked to patient-specific clinical variables, the framework may help clinicians explain the rationale for selected orthotic components. For example, clinicians may be able to contextualize recommendations for specific materials or structural supports using relevant patient characteristics, such as age or alignment-related measures. Such transparency may facilitate patient understanding of prescription decisions and support shared decision-making. The hierarchical architecture further enhances clinical applicability by ensuring continuity of decision support under incomplete data conditions [57]. In routine outpatient settings, advanced biomechanical assessments are often unavailable because of time or resource constraints. By adapting its decision pathway according to information availability, the CDSS maintains usability across diverse clinical environments and may help reduce dependence on specialized biomechanical assessments.

From an interface design perspective, the observed Top-K recommendation patterns have direct implications for how decision support outputs should be presented. A concise ranked shortlist may provide efficient initial guidance while minimizing alert fatigue and supporting clinician trust. At the same time, presenting a broader set of ranked recommendations may help clinicians review additional potentially relevant orthotic components in complex multicomponent prescriptions. In a practical deployment scenario, the CDSS could present clinicians with a ranked shortlist of recommended orthotic components together with component-specific probability estimates, allowing clinicians to review, accept, modify, or reject individual recommendations before final prescription.

### Limitations

Several limitations should be considered when interpreting these findings. First, clinician prescriptions in this study were derived from routine outpatient practice rather than from standardized consensus labeling or guideline-based adjudication. As a result, the training data may reflect interclinician variability as well as site-specific prescribing behaviors or implicit institutional biases. The model may therefore reflect prevailing institutional prescribing patterns rather than universally optimal or guideline-based prescription strategies. Future multicenter studies and external validation will be essential to enhance the generalizability and mitigate such effects.

Second, the data were collected over a historical period and may not fully capture temporal changes in orthotic materials, clinical guidelines, or prescribing practices. Longitudinal evaluation and periodic model updating will therefore be necessary to ensure the continued relevance of the CDSS as practice paradigms evolve.

Third, clinician prescriptions were used as a pragmatic reference standard, whereas downstream patient-centered outcomes—such as symptom improvement, functional gains, or long-term adherence—were not directly evaluated. Agreement with historical prescribing patterns does not necessarily imply improved clinical effectiveness. Although the model aligns with current expert practice, prospective studies will be required to determine whether CDSS-assisted prescriptions translate into improved clinical outcomes.

Fourth, although patient-level grouped cross-validation was implemented to prevent information leakage during model development and evaluation, the dataset originated from a single institution. Consequently, patient characteristics, clinical workflows, and prescribing patterns may differ across institutions, potentially limiting external generalizability.

Fifth, advanced biomechanical measurements were obtained selectively according to clinical judgment rather than through a standardized assessment protocol. As a result, the Level 8 complete-case subset may represent a clinically distinct subgroup, potentially limiting the generalizability of findings derived from this subset.

Sixth, imaging data such as radiographs or magnetic resonance imaging were not included in the present modeling framework. In some complex or structural deformities, orthotic prescription decisions may rely on anatomical information that cannot be fully captured through physical examination and biomechanical measurements alone. Therefore, the applicability of the current CDSS may be limited in clinical scenarios requiring imaging-informed decision-making.

Seventh, although missing values were retained as NaN and handled using the native missing-value processing capability of the tree-based models evaluated in this study, the mechanisms underlying missingness were not explicitly modeled. Because advanced biomechanical measurements were obtained selectively according to clinical judgment rather than at random, missingness itself may carry clinically relevant information. In addition, alternative missing-data handling strategies were not systematically evaluated in the present study. Consequently, the potential impact of different missing-data handling approaches on model calibration, feature-importance estimates, and model interpretability remains uncertain. Future studies should investigate whether incorporating explicit missingness indicators or other missingness-aware modeling strategies can further improve predictive performance and interpretability.

Eighth, although the dataset spanned a 6-year period (2015‐2020), model evaluation relied on cross-validation rather than temporal holdout validation. Therefore, the robustness of the proposed CDSS against time-dependent shifts in clinical practice was not explicitly assessed.

Ninth, several prescription components had extremely low prevalence, which likely contributed to reduced predictive performance and limited clinical utility for minority labels. In particular, some rare prescription components demonstrated very low AUPRC and PPV values despite acceptable AUROC estimates, indicating that discrimination performance alone may overestimate real-world predictive usefulness under severe class imbalance. Therefore, predictions for these components should be interpreted cautiously and are not yet sufficient to support independent clinical decision-making. In addition, several low-prevalence components exhibited substantial variability across cross-validation folds, particularly for PPV estimates. For some labels, the SD was large relative to the mean estimate, indicating limited stability of positive prediction performance. This pattern likely reflects severe class imbalance and the limited number of positive cases available for training and evaluation. Accordingly, performance estimates for these minority-label components should be interpreted together with their variability measures rather than relying solely on mean values. Although prevalence-sensitive metrics such as AUPRC and PPV were included to provide complementary performance assessment, explicit imbalance mitigation strategies—including resampling, cost-sensitive learning, class weighting, or synthetic minority augmentation—were not evaluated in the present study because the primary objective was to assess model feasibility using real-world prescription distributions across all clinically relevant orthotic components. Whether targeted imbalance mitigation strategies can improve prediction of rare prescription labels while preserving the clinical representativeness of real-world prescribing distributions remains an important area for future investigation.

Tenth, the conservative, safety-oriented threshold calibration strategy adopted in this study used component-specific thresholds to prioritize high specificity and minimize over-recommendation. Although this approach reduced the likelihood of false-positive component recommendations, it also substantially reduced sensitivity for several prescription components and may result in the omission of clinically relevant components in certain contexts. Consequently, threshold-calibrated outputs should be interpreted as high-confidence recommendations rather than comprehensive prescription suggestions. In clinical practice, clinician review remains essential to identify omitted or context-specific components before final prescription decisions. Adaptive or context-specific calibration strategies that balance sensitivity and specificity according to clinical context and prescription risks represent an important direction for future research.

Eleventh, although the hierarchical framework was designed to reflect routine clinical measurement availability, alternative modeling strategies, such as unified models with explicit missingness indicators or probabilistic data fusion approaches, were not systematically benchmarked in the present study. Therefore, although the proposed hierarchical framework demonstrated improved predictive performance when additional biomechanical measurements were available, whether this architecture provides advantages beyond workflow compatibility compared with alternative missing-data modeling strategies remains to be established.

Finally, although feature importance analysis provided clinically interpretable predictive patterns, these findings reflect predictive associations rather than causal relationships. In addition, the current framework provides only aggregate-level explanations and does not yet support case-specific explanation or uncertainty-aware recommendation outputs. Further work is needed to improve transparency and to better characterize how recommendations are generated for individual patients.

### Future Directions

Prospective validation of the proposed CDSS within routine clinical workflows remains a critical next step, with particular emphasis on clinician-in-the-loop evaluations and patient-reported outcomes. Whether CDSS-assisted prescriptions translate into sustained functional recovery and symptom relief remains to be established through prospective clinical evaluation.

An important methodological extension involves the development of dynamic clinician–AI interaction frameworks. While the current system demonstrates strong performance using calibrated probability-based Top-K recommendations, future iterations could adopt continual learning paradigms in which clinician overrides or modifications are incorporated as informative feedback signals. Such approaches may enable adaptive decision support that remains aligned with evolving clinical practice patterns. Any deployment of continually updated models would require structured governance mechanisms, including performance monitoring, periodic recalibration, and clinician oversight.

Alternative approaches for handling heterogeneous clinical information, including unified modeling strategies with explicit missingness indicators and probabilistic data fusion frameworks, warrant further evaluation. In addition, the current component-wise modeling strategy could be extended through multilabel architectures that explicitly capture dependencies among prescription components, such as classifier chains, structured output learning, or neural models.

Improving prediction performance for low-prevalence prescription components remains an important methodological challenge. Potential directions include dedicated class imbalance mitigation strategies, such as resampling, cost-sensitive learning, class weighting, or synthetic minority augmentation, as well as calibration approaches that dynamically balance sensitivity and specificity according to component-specific clinical risks and decision contexts. Recent work in gait-related ML has demonstrated that data balancing and generative augmentation strategies can improve classification performance in rare and highly imbalanced clinical datasets [58].

Future work may also investigate approaches for case-specific explanation and uncertainty communication, including instance-level feature attribution methods (eg, Shapley Additive Explanations [SHAP]) or uncertainty-aware recommendation outputs. Such extensions could help clinicians better understand model recommendations and evaluate their reliability in routine orthotic care.

### Conclusions

This study developed and evaluated a hierarchical CDSS for foot orthosis prescription using routinely collected outpatient clinical data from a large single-institution cohort. The proposed framework modeled prescription as a multilabel, component-based decision problem and combined probability-calibrated Top-K recommendations with component-specific safety-oriented thresholds. The hierarchical structure enabled decision support under varying levels of information availability in routine clinical practice while demonstrating improved predictive performance when additional biomechanical measurements were available.

The system provides component-wise recommendations intended to support, rather than replace, clinician judgment in routine orthotic care. By combining high-confidence threshold-calibrated recommendations with ranked Top-K outputs, the framework may support clinician-in-the-loop decision-making while reducing the likelihood of unnecessary component recommendations. Further prospective validation is required to establish its clinical utility and evaluate its performance in real-world orthotic care.

## Supplementary material

10.2196/92831 Multimedia Appendix 1 Additional tables.

## Abbreviations

AFO: ankle-foot orthosis
AUPRC: area under the precision-recall curve
AUROC: area under the receiver operating characteristic curve
CDSS: clinical decision support system
DECIDE-AI: Developmental and Exploratory Clinical Investigations of Decision support systems driven by AI
HGB: HistGradientBoosting
LightGBM: Light Gradient Boosting Machine
ML: machine learning
NaN: not a number
PPV: positive predictive value
RCSP: resting calcaneal stance position
ROM: range of motion
SHAP: Shapley Additive Explanations
STJ: subtalar joint
XGBoost: Extreme Gradient Boosting
