# Supplementary material for: A Hierarchical Machine Learning–Based Framework for Clinical Decision Support in Foot Orthosis Prescription: Algorithm Development and Validation Study
Source: JMIR Med Inform. 2026 Sep 22;14:e92831. doi: 10.2196/92831 (PMC13597017; doi:10.2196/92831)
Supplement: Supplementary file 1 [file medinform-v14-e92831-s001.docx]

Table S1. Component-specific thresholds, sensitivity, and specificity at the selected safety-oriented operating points for the Level 8 hierarchical model in the complete-case subset (n=3717).

| Label | Component | Positive cases, n (%) | Selected threshold | Sensitivity | Specificity |
| --- | --- | --- | --- | --- | --- |
| P1 | Forefoot extension | 3248 (87.4) | 0.98 | 0.382 | 0.953 |
| P2 | Poron base material | 537 (14.4) | 0.44 | 0.371 | 0.951 |
| P3 | Multi-density foam | 2226 (59.9) | 0.82 | 0.192 | 0.952 |
| P4 | Cork-based foundation | 878 (23.6) | 0.46 | 0.167 | 0.952 |
| P5 | Polyethylene base material | 454 (12.2) | 0.35 | 0.328 | 0.952 |
| P6 | Metatarsal dome | 392 (10.5) | 0.35 | 0.245 | 0.956 |
| P7 | Heel poron cushion | 2406 (64.7) | 0.86 | 0.217 | 0.951 |
| P8 | Medial inverted balance posting | 629 (16.9) | 0.36 | 0.215 | 0.952 |
| P9 | Medial forefoot flange | 152 (4.1) | 0.13 | 0.164 | 0.960 |
| P10 | Lateral flange | 100 (2.7) | 0.08 | 0.480 | 0.953 |
| P11 | Gait plate | 450 (12.1) | 0.33 | 0.400 | 0.952 |
| P12 | Reverse gait plate | 12 (0.3) | 0.01 | 0.250 | 0.977 |
| P13 | UCBL(University of California Biomechanics Laboratory orthosis) | 26 (0.7) | 0.05 | 0.000 | 1.000 |
| P14 | Navicular support | 419 (11.3) | 0.34 | 0.616 | 0.952 |
| P15 | Kinetic wedge | 72 (1.9) | 0.09 | 0.194 | 0.962 |

Table S2. Baseline characteristics of the full cohort and the Level 8 complete-case subset used to assess potential selection bias.

| Variable | Full cohort  (n = 6462) | Level 8 complete-case subset  (n = 3717) | P value |
| --- | --- | --- | --- |
| Demographic characteristics | | | |
| Age, mean (SD) | 16.60 (17.07) | 19.84 (18.81) | <.001 |
| Male, n (%) | 3144 (48.7) | 1719 (46.2) | 0.020 |
| Female, n (%) | 3318 (51.3) | 1998 (53.8) | 0.020 |
| Alignment characteristics | | | |
| L_RCSP, mean (SD) | -3.43 (4.73) | -3.17 (4.85) | 0.009 |
| R_RCSP, mean (SD) | -2.63 (4.55) | -2.20 (4.65) | <0.001 |
| Prescription component prevalence | | | |
| P1, n (%) | 5683 (87.9) | 3249 (87.4) | 0.446 |
| P2, n (%) | 747 (11.6) | 538 (14.5) | <.001 |
| P3, n (%) | 3731 (57.7) | 2227 (59.9) | 0.034 |
| P4, n (%) | 2021 (31.3) | 879 (23.6) | <.001 |
| P5, n (%) | 617 (9.5) | 454 (12.2) | <.001 |
| P6, n (%) | 490 (7.6) | 392 (10.5) | <.001 |
| P7, n (%) | 3624 (56.1) | 2406 (64.7) | <.001 |
| P8, n (%) | 1115 (17.3) | 628 (16.9) | 0.663 |
| P9, n (%) | 330 (5.1) | 152 (4.1) | 0.023 |
| P10, n (%) | 221 (3.4) | 100 (2.7) | 0.049 |
| P11, n (%) | 835 (12.9) | 450 (12.1) | 0.246 |
| P12, n (%) | 35 (0.5) | 12 (0.3) | 0.157 |
| P13, n (%) | 110 (1.7) | 26 (0.7) | <.001 |
| P14, n (%) | 1206 (18.7) | 419 (11.3) | <.001 |
| P15, n (%) | 112 (1.7) | 72 (1.9) | 0.505 |

Values are presented as mean (SD) for continuous variables and n (%) for categorical variables. P values were calculated using independent t tests for continuous variables and chi-square tests for categorical variables.

Table S3. Hyperparameter search space used for Optuna optimization of the HistGradientBoosting classifier.

| Hyperparameter | Search range | Sampling distribution |
| --- | --- | --- |
| max_iter | 500–1500 | Integer |
| max_depth | 3–8 | Integer |
| learning_rate | 0.001–0.1 | Log-uniform |
| l2_regularization | 0.001–10 | Log-uniform |
| max_bins | 100–255 | Integer |
| min_samples_leaf | 10–100 | Integer |

Table S4. Final component-specific hyperparameter configurations identified by Optuna using 5-fold stratified grouped cross-validation for the Level 4 and Level 8 hierarchical models. Hyperparameter optimization was performed separately for each prescription component, and the configuration achieving the highest mean AUROC across grouped cross-validation folds was selected as the final optimized parameter set.

| Level 4 | | | | | | | |
| --- | --- | --- | --- | --- | --- | --- | --- |
| Label | Component | max_iter | max_depth | learning_rate | l2_regularization | max_bins | min_samples_leaf |
| P1 | Forefoot extension | 1323 | 5 | 0.003 | 0.065 | 157 | 31 |
| P2 | Poron base material | 1230 | 3 | 0.003 | 0.070 | 127 | 51 |
| P3 | Multi-density foam | 773 | 4 | 0.004 | 0.003 | 242 | 74 |
| P4 | Cork-based foundation | 510 | 5 | 0.008 | 0.002 | 251 | 47 |
| P5 | Polyethylene base material | 763 | 4 | 0.007 | 0.138 | 176 | 93 |
| P6 | Metatarsal dome | 882 | 3 | 0.002 | 0.002 | 115 | 61 |
| P7 | Heel poron cushion | 763 | 5 | 0.007 | 1.712 | 223 | 33 |
| P8 | Medial inverted balance posting | 989 | 4 | 0.014 | 8.921 | 145 | 43 |
| P9 | Medial forefoot flange | 1028 | 3 | 0.002 | 0.001 | 255 | 79 |
| P10 | Lateral flange | 1069 | 5 | 0.004 | 0.152 | 186 | 58 |
| P11 | Gait plate | 1207 | 3 | 0.011 | 2.256 | 194 | 23 |
| P12 | Reverse gait plate | 819 | 6 | 0.003 | 0.004 | 134 | 17 |
| P13 | UCBL(University of California Biomechanics Laboratory orthosis) | 1137 | 6 | 0.009 | 6.701 | 106 | 39 |
| P14 | Navicular support | 951 | 6 | 0.004 | 0.031 | 109 | 94 |
| P15 | Kinetic wedge | 1176 | 3 | 0.003 | 0.037 | 241 | 84 |
| Level 8 | | | | | | | |
| Label | Component | max_iter | max_depth | learning_rate | l2_regularization | max_bins | min_samples_leaf |
| P1 | Forefoot extension | 1062 | 3 | 0.007 | 5.081 | 104 | 77 |
| P2 | Poron base material | 540 | 8 | 0.008 | 7.627 | 222 | 99 |
| P3 | Multi-density foam | 1268 | 5 | 0.003 | 0.004 | 181 | 32 |
| P4 | Cork-based foundation | 1348 | 6 | 0.004 | 0.950 | 131 | 85 |
| P5 | Polyethylene base material | 504 | 7 | 0.006 | 8.328 | 251 | 100 |
| P6 | Metatarsal dome | 762 | 4 | 0.005 | 0.100 | 255 | 11 |
| P7 | Heel poron cushion | 1256 | 5 | 0.004 | 0.462 | 126 | 69 |
| P8 | Medial inverted balance posting | 508 | 3 | 0.021 | 3.414 | 127 | 83 |
| P9 | Medial forefoot flange | 1232 | 4 | 0.003 | 0.037 | 172 | 55 |
| P10 | Lateral flange | 1309 | 3 | 0.002 | 0.023 | 128 | 24 |
| P11 | Gait plate | 1135 | 4 | 0.004 | 0.011 | 206 | 28 |
| P12 | Reverse gait plate | 838 | 3 | 0.004 | 0.004 | 229 | 30 |
| P13 | UCBL(University of California Biomechanics Laboratory orthosis) | 957 | 8 | 0.002 | 0.248 | 196 | 85 |
| P14 | Navicular support | 1237 | 3 | 0.006 | 0.012 | 229 | 45 |
| P15 | Kinetic wedge | 1231 | 3 | 0.001 | 3.570 | 239 | 23 |
